# Belief in Communism and Theory of Mind

**DOI:** 10.3389/fpsyg.2021.697251

**Published:** 2021-08-05

**Authors:** Outong Chen, Fang Guan, Yu Du, Yijun Su, Hui Yang, Jun Chen

**Affiliations:** ^1^School of Psychology, South China Normal University, Guangzhou, China; ^2^Normal College & School of Teacher Education, Qingdao University, Qingdao, China; ^3^Guangdong Key Laboratory of Mental Health and Cognitive Science, South China Normal University, Guangzhou, China; ^4^Center for Studies of Psychological Application, South China Normal University, Guangzhou, China; ^5^Department of Psychology, Tsinghua University, Beijing, China

**Keywords:** belief in communism, regional homogeneity, resting state functional connectivity, theory-of-mind, neural basis

## Abstract

A belief in communism refers to the unquestionable trust and belief in the justness of communism. Although former studies have discussed the political aim and social value of communism, the cognitive neural basis of a belief in communism remains largely unknown. In this study, we determined the behavioral and neural correlates between a belief in communism and a theory of mind (ToM). For study 1, questionnaire scores were measured and for study 2, regional homogeneity (ReHo) and resting-state functional connectivity (rsFC) were used as an index for resting-state functional MRI (rs-fMRI), as measured by the Belief in Communism Scale (BCS). The results showed that a belief in communism is associated with higher ReHo in the left thalamus and lower ReHo in the left medial frontal gyrus (MFG). Furthermore, the results of the rsFC analysis revealed that strength of functional connectivity between the left thalamus and the bilateral precuneus is negatively associated with a belief in communism. Hence, this study provides evidence that spontaneous brain activity in multiple regions, which is associated with ToM capacity, contributes to a belief in communism.

## Introduction

A belief is defined as a state of mind in which a person places trust or confidence in the existence or justness of a person or thing, with or without supporting empirical evidence (Zalta et al., 2008). Thus, a belief in communism refers to the trust and belief in the justness of communism. Communism is an important political and social ideology, as it refers to the philosophical, social, political, and economic ideology and movement to establish a communist society (Bukharin and Preobrazhenskii, 1921); however, beyond this, communism is also a broader cultural system (Paretskaya, 2010). A communist society is a socioeconomic order structured upon the common ownership of the means of production and the absence of social classes, money, and the state (Kurian and Boryczka, 2010).

A belief in communism is highly related to social relations and cooperation (Wang, 2002). To achieve a common goal and fulfill a common need, conspecifics must be able to cooperate and establish emotional contact (not necessarily involving physical contact), which are essential behaviors for social animals (Proverbio et al., 2011). These social abilities have been suggested to be correlated with theory of mind (ToM) abilities. The ToM is a social cognitive construct that refers to the ability to make high-level inferences about the mental states of an individual as well as those of others (i.e., thoughts, feelings, desires, and beliefs) (Premack and Woodruff, 1978). Such ToM abilities are fundamental to human social function (Elliott et al., 2006). Former studies have revealed an association between the development of ToM abilities and social cognition at the behavioral level. Hughes and Leekam (2004) suggested a strong association between the development of ToM abilities and social interactions among children, including how and with whom they interact. Astington and Jenkins (1995) suggested that capacity of an individual for social understanding, social interaction, and some aspects of language are related to their ToM abilities. Therefore, we hypothesized that a belief in communism and ToM are correlated at the behavioral level because they share a similar social cognitive function.

Similarly, at the neural level, most previous neuroimaging studies have revealed that processing of social cognition is correlated with the major functioning of the ToM brain network (Schilbach et al., 2008). The ToM network refers to large areas of the brain that are related to ToM brain functions, such as the medial pre-frontal cortex, posterior cingulate cortex, right anterior superior temporal sulcus, medial precuneus, and posterior cingulate cortex (Kana et al., 2015). A functional MRI (fMRI) study revealed that brain activation in the ToM network is correlated with responses of the participants in a public goods game involving interpersonal relationships and cooperation (Špiláková et al., 2019). Furthermore, Lissek et al. (2008) found that parts of the ToM network, such as the pre-frontal cortex, play an important role in complex social interactions. These social interaction skills are also key to a belief in communism, since social cognition abilities, such as social relations and cooperation, are necessary to achieve a common goal and fulfill a common need (Wang, 2002; Proverbio et al., 2011). However, since empirical evidence from neural imaging studies is lacking, we can only indirectly hypothesize that a belief in communism may be correlated with ToM at the neural level. Given the fact that ToM is not a monolithic process but composed of complex cognitive as well as affective processing (Shamay-Tsoory et al., 2006), we assume that multiple brain regions of the ToM network may be involved in the neural correlates of a belief in communism.

Decades after the establishment of communism, its political aim and social values have been well-discussed; however, to our knowledge, no studies have systematically addressed the psychological basis of a belief in communism. Therefore, we designed two studies to reveal the psychological basis of a belief in communism at the behavioral level and the neural level. Study 1 aimed to establish an association between a belief in communism and ToM at the behavioral level. Belief in communism was measured using the Belief in Communism Scale (BCS) (McFarland, 1998). In accordance with previous research on ToM (Arous et al., 2016), ToM abilities were measured using two different questionnaires: the Interpersonal Reactivity Index (IRI) (Aketa, 1999) and the Questionnaire of Cognitive and Affective Empathy (QCAE) (Reniers et al., 2011). To match the language and cultural background of the participants, revised versions of IRI (Zhang et al., 2010) and QCAE (Wang and Su, 2019) were used. The IRI and the QCAE serve as two independent indicators of ToM abilities. We predicted that a belief in communism would be positively correlated with ToM. Study 2 aimed to reveal an association between a belief in communism and ToM abilities at the neural level. We measured regional homogeneity (ReHo) across the whole brain and further adopted the resting-state functional connectivity (rsFC) of the resting-state fMRI (rs-fMRI) signals to investigate brain regions and networks potentially associated with a belief in communism. An identical measurement of the BCS from study 1 was adopted as the behavioral measurement. We predicted that a belief in communism would be associated with brain regions of the ToM brain network.

## Study 1

### Materials and Methods

#### Participants

A total of 382 participants (316 women, 66 men) recruited locally throughout the university campus network participated in this study. Participants aged from 23 to 55 (*M* = 37.79, *SD* = 6.55), and <1% of data was missing, which was handled using mean imputation (Little and Rubin, 2002). All participants provided written informed consent prior to participating in the study, and all procedures were performed in accordance with the Declaration of Helsinki.

#### Measures

***Belief in Communism Scale*. **The BCS was used to assess the degree of the belief of the participants in communism. The BCS consists of 38 items divided into 4 dimensions: fundamentalism (items 1–8), intrinsic (items 9–19), extrinsic (items 20–29), and quest orientation (item 30–38) dimensions of a belief in communism. The items consist of statements such as: “the principles worked out by Marx and Lenin show us the way to solve today's problems,” and “every communist must contribute to the development of communism in other countries.” Participants respond to these items on a 5-point Likert scale (1 = *strongly disagree* and 5 = *strongly agree*). To ensure the reliability and validity of the Chinese translation of the scale in this study, two English major specialists were invited to translate the BCS to Chinese. After the translation, 10 students evaluated the semantic clarity, cultural appropriateness, and grammatical aspects of each item. The translated version was then discussed between the members of the lab, after which the final version was established. The total score is summed and higher scores represent a stronger belief in communism. This measure showed adequate internal consistency in this study (full-scale Cronbach's α = 0.74).

***Interpersonal Reactivity Index*. **The IRI was used to evaluate ToM abilities. The IRI evaluates four domains related to ToM and mentalizing: The perspective-taking (PT) scale assesses the cognitive tendency to adopt the psychological perspective of others in everyday life (e.g., “I sometimes try to understand my friends better by imagining how things look from their perspective”). The fantasy scale (FS) assesses the tendency to imaginatively identify with the feelings and actions of fictional characters (e.g., “I really get involved with the feelings of the characters in a novel”). The final two scales measure emotional reactions in response to the distress of others. The empathetic concern (EC) scale assesses feelings of warmth, compassion, and concern for others in distress (e.g., “I often have tender, concerned feelings for people less fortunate than me”), and the personal distress (PD) scale measures self-focused feelings of personal anxiety and discomfort in response to the misfortune of others (e.g., “when I see someone who badly needs help in an emergency, I go to pieces”). Participants respond to each item using a 5-point Likert-type scale ranging from (0) “*does not describe me well*” to (4) “*does describe me very well*.” This measure showed adequate internal consistency in this study (full-scale Cronbach's α = 0.71).

***Questionnaire of Cognitive and Affective Empathy*. **The QCAE is an empathy measure composed of 31 items measuring cognitive (19 items; e.g., “I am good at predicting how someone will feel”) and affective (12 items; e.g., “I am inclined to get nervous when others around me seem to be nervous”) dimensions of empathy, which is also a core dimension of a capacity for ToM. Participants rated their responses on a 4-point Likert scale, which range from *strongly agree* (1) to *strongly disagree* (4). We summed the item responses to create composite scores for the total scale (Cronbach's α = 0.81), with high scores indicating increased levels of empathy.

#### Procedure

After reading and signing a brief informed consent form, the participants were asked to complete the questionnaires, which included the BCS, IRI, and QCAE, as well as some other unrelated questionnaires to prevent the participants from guessing the purpose of the study. No post-study inquiries indicated that any of the participants suspected the actual purpose of the study. After the questionnaires, the participants completed a brief demographics survey, which included gender, age, and party member status. In the politics status section, participants were asked to indicate if they were members of the communist party. All the questionnaires were anonymous. At the end of the study, all participants were thanked for participating and debriefed.

#### Results

***Correlational Analysis*. **Table 1 contains univariate statistics and bivariate correlations for all study variables. The results indicated that BCS was positively correlated with both the IRI (*r* = 0.17, *p* < 0.001, 95% CI [0.07, 0.27]) and the QCAE (*r* = 0.30, *p* < 0.001, 95% CI [0.20, 0.39]). In addition, the IRI and the QCAE, as individual indicators of ToM, were significantly correlated with each other (*r* = 0.46, *p* < 0.001, 95% CI [0.38, 0.54]). We also conducted a similar correlational analysis with gender, age, religion, and politics status controlled as covariables. The second analysis indicated that BCS was still positively correlated with the IRI (*r* = 0.19, *p* < 0.001) and the QCAE (*r* = 0.28, *p* < 0.001). Thus, the present correlative results confirmed the hypothesis of study 1.

**Table 1:** Means, SDs, and correlation matrix among the variables in study 1.

**Variables**	**Mean**	**SD**	**1**	**2**	**3**	**4**	**5**
1. Gender	—	—	—				
2. Age	38.41	6.06	0.07	—			
3. BCS	102.95	11.16	0.07	0.10	—		
4. IRI	80.90	9.75	−0.15^**^	−0.01	0.17^***^	—	
5. QCAE	87.41	10.37	0.11^*^	0.08	0.30^***^	0.46^***^	—

*SD, standard deviation; BCS, Belief in Communism Scale; IRI, Interpersonal Reactivity Index; QCAE, Questionnaire of Cognitive and Affective Empathy*

*** *p < 0.001;*

** *p < 0.01;*

* *p < 0.05*.

## Study 2

### Materials and Methods

#### Participants

According to a recent discussion about sample size and reliability for correlational neuroimaging studies (Chen et al., 2018), we recruited 50 healthy adults from a university (12 men; *M*_age_ = 20.60 years; *SD*_age_ = 0.43). All participants were right-handed with normal or corrected-to-normal vision. None of the participants had a history of mental or neurological illness. This study was approved by the Institutional Review Board of the university. All participants provided written informed consent before participating in the study.

#### Behavioral Assessments

An identical measure of the BCS from study 1 was adopted to measure the belief of the participants in communism in study 2. This measure also showed adequate internal consistency in this study (full-scale Cronbach's α = 0.79).

#### MRI Data Acquisition

The rs-fMRI scan was performed on a 3.0-T scanner (Siemens Magnetom Trio, A Tim System, South China Normal University, Guangzhou, China.) equipped with a 12-channel phased-array head coil at the Brain Imaging Center. Resting-state scanning consisted of 240 contiguous echo-planar imaging (EPI) images (TR = 2,000 ms; TE = 30 ms; flip angle = 90°; slices = 32; matrix = 64 × 64; FOV = 220 × 220 mm^2^; thickness/gap = 3.5/0.8 mm). Simultaneously, high-resolution T1-weighted structural images were collected using an MPRAGE sequence (TR/TE = 1,900/2.52 ms; flip angle = 9; matrix = 256 × 256; slice thickness = 1.0 mm; sagittal slices = 176). During resting-state scanning, participants were instructed to remain awake with their eyes closed and to not think about anything in particular.

#### Data Preprocessing

The Data Processing Assistant for rs- fMRI software (DPARSF; http://www.restfmri.net/forum/DPARSF) (Yan and Zang, 2010) on the MATLAB platform (MathWorks, Natick, MA, USA) was the main toolbox used for preprocessing the functional imaging data, following a previously established protocol (Xiang et al., 2016):

1. The quality of the scanning image was checked for each participant and the DICOM file format was converted to a NIfTI file format.
2. Images from the first 10 time points were discarded to ensure steady-state magnetization. The remaining 230 volumes were included in the final analysis.
3. Slice timing and head motion correction were performed to correct slice order and head motion effects, respectively.
4. After obtaining a mean functional image for each participant, the mean functional images were registered to the structural images and were subsequently segmented as gray matter, white matter, and cerebrospinal fluid.
5. Each functional image was normalized to the standard Montreal Neurological Institute space in 3 × 3 × 3 mm^3^ voxel sizes with the application of the parameters obtained during segmentation.
6. The East Asian brain template provided by the Statistical Parametric Mapping software (SPM 12); Wellcome Department of Cognitive Neurology, London, United Kingdom; www.fil.ion.ucl.ac.uk/spm) was used for normalization.
7. The linear trends were removed and the images were temporally band-pass filtered (0.01–0.08 Hz) to reduce low-frequency drift and high-frequency noise (Biswal et al., 1995).

#### Statistical Analyses

***ReHo Analysis*. **We measured the ReHo of the time series for a given voxel with the nearest 26 neighboring voxels in a voxel-wise manner using Kendall's coefficient of concordance (KCC). The formula used to calculate the KCC has been reported elsewhere (Zang et al., 2004). The procedures used to obtain individual ReHo maps were implemented using DPARSF. To obtain the ReHo Z-score map for each participant, we subtracted the global mean ReHo and divided it by the SD. The data were then smoothed with an 8-mm full width at half maximum (FWHM) Gaussian kernel. Data analysis was performed using the Data Processing and Analysis of Brain Imaging, DPABI, v3.0, (http://rfmri.org/DPABI; Yan et al., 2016) toolbox. For the whole-brain analyses, a correlational model was applied using the BCS score as the variable of interest to identify regions where ReHo was correlated with individual differences at the level of a belief in communism after controlling for possible confounding variables, such as age and gender. Spatial smoothing was performed with an 8 mm FWHM Gaussian kernel on the ReHo maps. Cluster correction using Gaussian random-field theory was then applied to the threshold voxels to correct for multiple comparisons (Worsley et al., 1996). A cluster probability of *p* < 0.05 was considered significant for activations.

***Functional Connectivity Analysis*. **Furthermore, we conducted a functional connectivity analysis to examine whether the regions that were observed in the ReHo-behavioral correlation analysis worked in concert with other regions as a network that was correlated with the BCS. The regions that were significantly correlated with the BCS in previous analyses were used as seed regions of interest to compute functional connectivity between the seeds and other voxels in the whole brain. A correlation analysis was then conducted between the specific functional connectivity strength and the BCS score to examine whether any specific connections within these networks were associated with the BCS. The rsFC analysis was performed using DPABI. Before the analysis, nuisance covariates, including white matter signals, cerebrospinal fluid signals, global mean signals, and Friston 24 motion parameters (Friston et al., 1996), were regressed out. First, the mean time series of the seeds were calculated for each participant and correlated with the time series of all other voxels in the gray matter, as previously described. Second, the correlation maps produced in this analysis were then converted to Z-maps using Fisher's r-to-z transformation. Last, a one-sample *t*-test was conducted to identify the regions that were significantly connected with the seeds (*p* < 0.01, corrected). The significant regions were then defined as masks, and the rsFC-behavior correlation analyses were conducted in these masks to examine which functional connectivity strength was correlated with the BCS score.

#### Results

***Behavioral Data***. Table 2 shows the mean, SD, and range of the BCS. No significant gender difference in the BCS score was observed, *t*(48) = 0.29, *p* = 0.77, Cohen's *d* = 0.10, 95% CI = [−6.19, 8.31], and no significant correlation between the BCS and age was observed, *r* = −0.10, *p* = 0.51, 95% CI = [−0.36, 0.19].

**Table 2:** Descriptive statistics of the BCS scores in study 2.

**Variables**	**Mean**	**SD**	**Range**
BCS	93.90	10.80	58–117

*SD, standard deviation; BCS, Belief in Communism Scale*.

**Figure 1:**
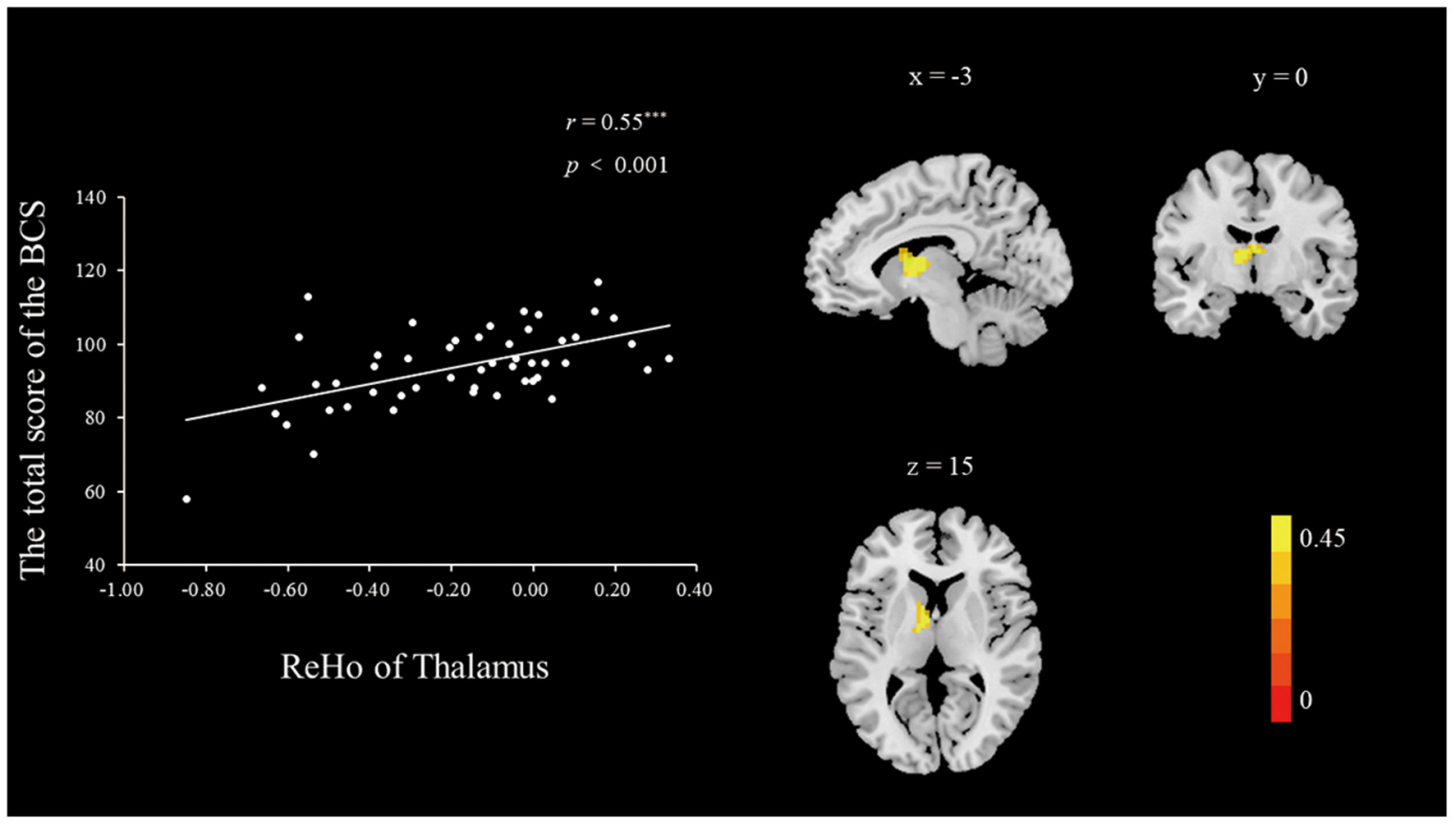
The brain regions with significant correlations between the ReHo and the BCS (right panel). The ReHo in the left thalamus was positively correlated with the BCS (left panel). The scatter plots depicted correlations between the ReHo in the left thalamus and individual differences in the BCS scores. Height and extent thresholds were set at *p* < 0.01, corrected.

**Figure 2:**
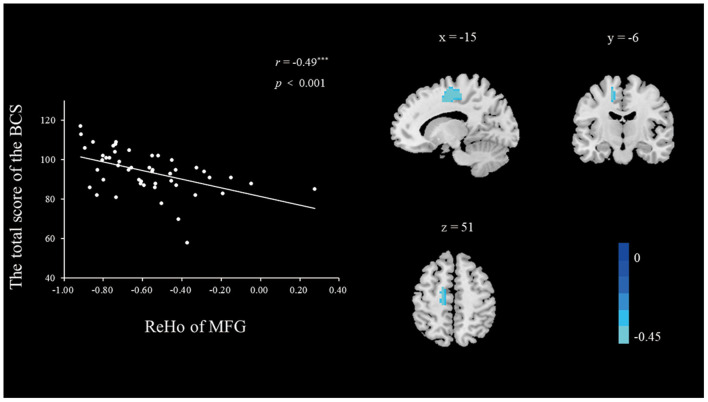
The brain regions with significant correlations between the ReHo and the BCS (right panel). The ReHo in the left MFG was negatively correlated with the BCS (left panel). The scatter plots depicted correlations between ReHo in the left MFG and individual differences in the BCS scores. Height and extent thresholds were set at *p* < 0.01, corrected.

***ReHo During the Resting State*. **To explore the neural correlates of a belief in communism, we correlated the BCS scores with the ReHo value of each voxel across the whole brain. A correlation analysis was conducted between the standardized ReHo values and the total BCS score with age and gender as nuisance covariates. As shown in Table 3 and Figures 1, 2, the BCS was positively correlated with ReHo in the left thalamus (*r*_peak_ = 0.46, *r*_cluster_ = 0.55, *p* < 0.001) and negatively correlated with ReHo in the left medial frontal gyrus (MFG; *r*_peak_ = −0.49, *r*_cluster_ = −0.49, *p* < 0.001). No other significant relationships were observed.

**Table 3:** Significant associations between brain regions and the BCS.

**Brain region**	**Side**	**BA**	**MNI**			**Voxel size**	**Peak *R***
			***X***	***y***	***Z***		
Thalamus	L		−3	0	15	196	0.46^***^
MFG	L	6	−15	−6	51	160	−0.49^***^

*MNI, Montreal Neurological Institute; L, left; BA, Brodmann area; MFG, medial frontal gyrus*

*** *p < 0.001*.

**Figure 3:**
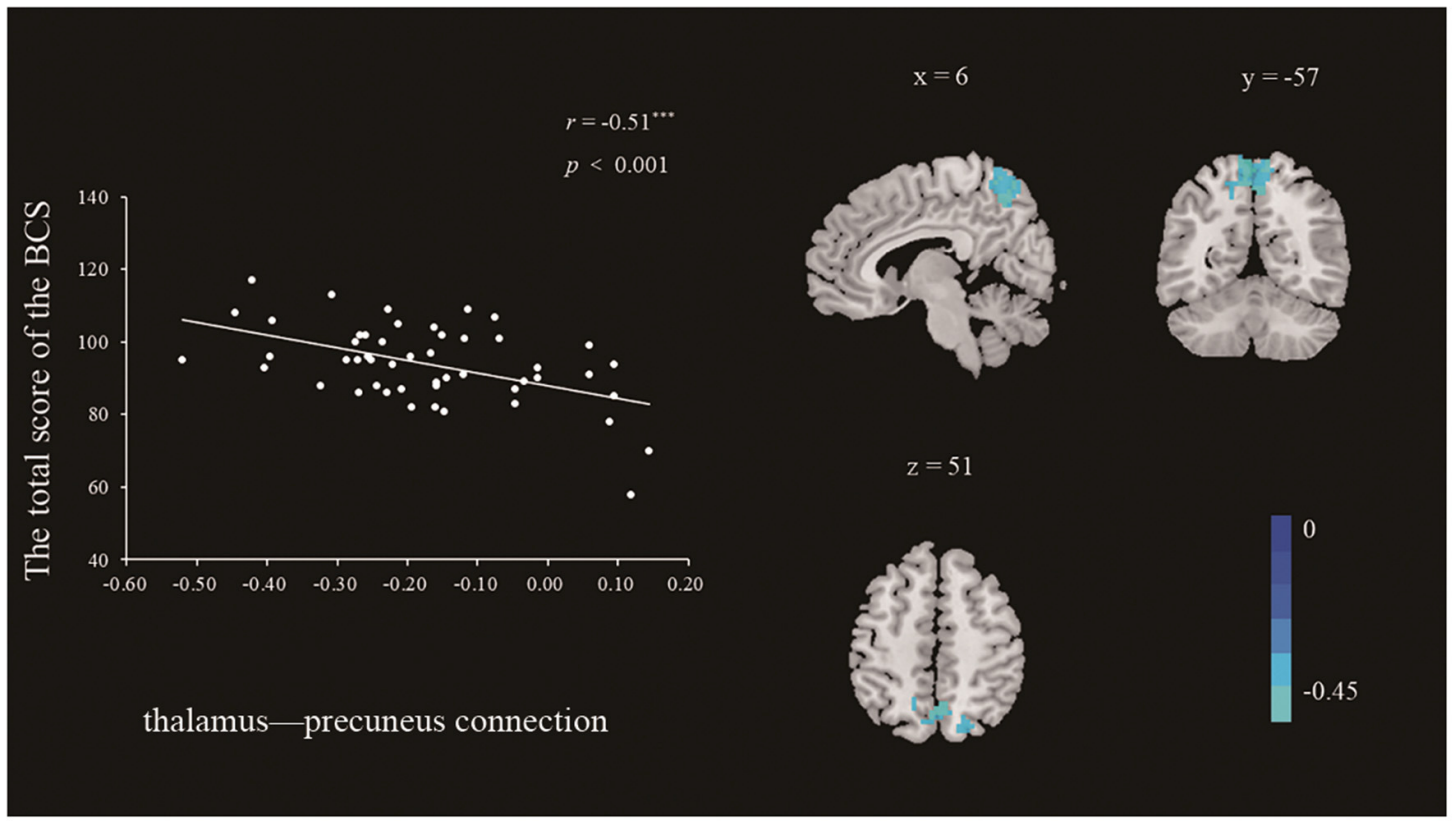
Clusters whose functional connectivity strengths with the left thalamus (seed) were significantly associated with the BCS (right panel). Color bars represent *R*-values. The functional connectivity strength between the left thalamus and bilateral precuneus was negatively correlated with the BCS (left panel). The scatter plots depicted correlations between functional connectivity strength and individual differences in the BCS scores. The threshold was set at *p* < 0.01, corrected.

***Functional Network Associated With the BCS*. **After identifying the neural correlates of a dispositional belief in communism, we further examined whether the brain regions that had been identified in previous analyses functioned synergistically with other regions as the neural basis of a belief in communism. We performed a correlation analysis between the strength of functional connectivity and the BCS score with age and gender as nuisance covariates. The significant regions in the ReHo-behavior correlation analysis were used as seeds in the rsFC analysis. As shown in Table 4 and Figure 3, we found that strength of functional connectivity between the left thalamus and the bilateral post-central gyrus was significantly associated with the BCS (*r*_peak_ = −0.46, *r*_cluster_ = −0.51, *p* < 0.001). No other significant results were identified in this analysis.

**Table 4:** Regions for which the strength of the functional connectivity with the thalamus was significantly associated with the BCS.

**Brain regions**	**Side**	**BA**	**MNI**			**Voxel size**	**Peak *R***
			***X***	***y***	***Z***		
Precuneus	Bilateral	7	6	−57	51	251	−0.46^***^

*MNI, Montreal Neurological Institute; BA, Brodmann area*

*** *p < 0.001*.

**Figure 4:**
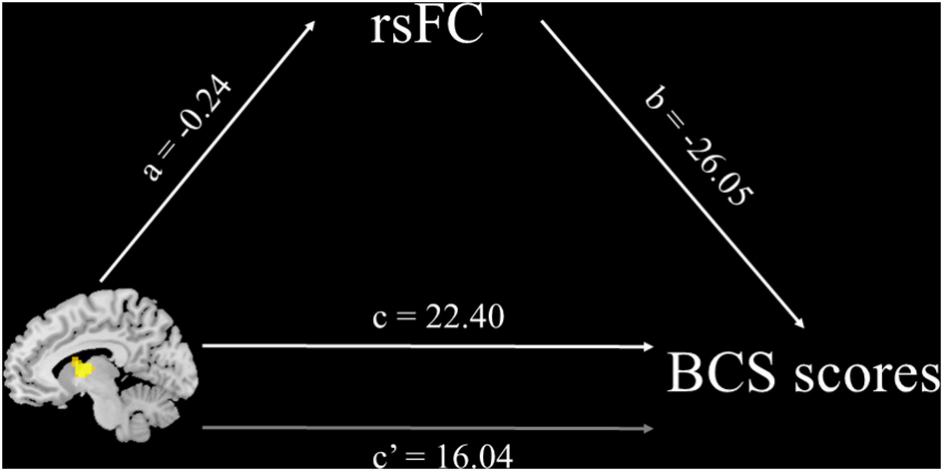
Mediation analysis. Path c is the total effect of ReHo in the left thalamus on BCS scores; path c′ is the direct effect of ReHo in the left thalamus on BCS scores after controlling for rsFC values of the thalamus–precuneus connection; the product of paths a and b (ab) is the indirect effect of ReHo in the left thalamus through rsFC values of the thalamus–precuneus connection on BCS scores. The standardized path coefficients are displayed on the path lines. Mediation analysis suggests that rsFC between the thalamus and the precuneus can account for the relationship between ReHo in the left thalamus and BCS scores.

***Mediating Effect of rsFC Between the Left Thalamus and the Precuneus*. **Following the previous analysis, we further conducted a mediating effect analysis to investigate whether the discovered rsFC could account for the relationship between ReHo in the left thalamus and the BCS scores. A typical mediation analysis was conducted using Hayes (2013) PROCESS 3.0 macro for SPSS (Model 4). The total effect of an independent variable X on a dependent variable Y (path c) was estimated, which included the direct effect of X on Y after controlling for mediator M (path c′) and the indirect effect of X on Y through M (see Figure 4). For this study, the ReHo in the left thalamus extracted from the resulting mask was the independent variable, the BCS score was the dependent variable, and the rsFC between the left thalamus and the precuneus from the previous section was the mediator. After controlling for age and gender, the mediation analysis showed that rsFC between the left thalamus and BCS partially mediated the association between ReHo in the left thalamus and the BCS score [c′ = 16.04, *p* < 0.01, refer to Figure 4]. The mediating effect accounted for 16% of the total effect and the rsFC between the left thalamus and BCS accounted for 28.39% of the variance in the BCS scores (*p* < 0.001).

## Discussion

In this study, we designed two studies to investigate the psychological basis of a belief in communism by revealing a unique relationship with ToM. Study 1 revealed a direct, positive relationship between a belief in communism and ToM abilities at the behavioral level. For study 2, ReHo-behavioral correlation analyses and seed-based rsFC analyses were conducted to investigate the neural correlates of a belief in communism. We found that individual differences in a belief in communism were reflected in the ReHo and rsFC. Specifically, a belief in communism was associated with ReHo in the left MFG and left thalamus. We further observed that strength of functional connectivity between the left thalamus and the bilateral precuneus was associated with individual differences in the belief of participants in communism. Furthermore, the exploratory mediation analysis showed that thalamus–precuneus circuit could account for the relationship between ReHo in the left thalamus and the BCS scores.

To achieve a common goal and fulfill a common need, which is the main tenant of communism (Bukharin and Preobrazhenskii, 1921), a capacity for social understanding, social interaction, and cooperation is needed (Wang, 2002). Interestingly, these abilities are closely correlated with ToM abilities (Astington and Jenkins, 1995). In one study, Sally and Hill (2006) used a prisoner's dilemma paradigm and asked participants to choose from two types of strategies: cooperate with each another for joint gain or compete for the own gain of the individual. The results indicated that option to cooperate was associated with higher ToM abilities. This study findings at the behavioral level were consistent with these studies. We concluded that ToM abilities may be the psychological basis of a belief in communism since people with high levels of a belief in communism also have greater ToM abilities.

Consistent with our hypothesis for study 2, a belief in communism was associated with the ToM brain network at the neural level. First, ReHo in the thalamus was identified to be positively associated with individual differences in the belief of participants in communism. The proposition of communism is characterized by social relations and cooperation (Wang, 2002). For example, a social study conducted in Romania has suggested that Romanians attach more importance to social relations and connections as a legacy of residual communism (Gavreliuc and Ciobotă, 2013), and ToM abilities are necessary to achieve the requirements of communist society with regard to social cognition and social values (Hughes and Leekam, 2004). At the neural level, the thalamus has been reported relatively less in association with the ToM network. However, former researchers have suggested that thalamus inputs neural signals to the temporoparietal junction (TPJ) and thus indirectly connects with the pre-frontal and temporal cortices (Decety and Lamm, 2007), which are involved in the ToM network. Most of the earlier experimental tasks related to ToM abilities have not involved actual social relationships and real-life outcomes. Thus, involvement of previously unreported areas, such as the thalamus, may represent brain regions that are uniquely involved in interaction, cooperation, and communication (Rilling et al., 2004). For example, in an fMRI study conducted by Reniers et al. (2014), the thalamus was activated during a task examining the neural substrates of ToM, empathy, and self-other differentiation involved in the adaptive understanding of the internal states of people. Based on their findings, the thalamus could be a positive indicator of ToM abilities. Thus, the positive relationship between ReHo of the thalamus and belief in communism in this study may reflect that higher levels of a belief in communism are associated with greater ToM abilities. Therefore, this result supported our main hypothesis that a belief in communism is positively associated with greater ToM abilities, which contribute to the characteristics of social interaction, cooperation, and communication representative of communism.

In contrast to the thalamus, ReHo of the MFG was identified to be negatively associated with the individual differences in the belief of participants in communism in this study. Previous studies have suggested that MFG is involved in the process of the “self,” (Liu et al., 2017) highlighting its involvement during self-construal. A study conducted by Li et al. (2018), for example, revealed that a measure of independent (vs. interdependent) self-construal is correlated with multiple brain regions, including the MFG. In that study, the MFG was found to be positively correlated with a more independent self-construal, which reversely indicates that MFG is negatively correlated with a more interdependent self-construal. Self-construal is a shared component of both a belief in communism and ToM. Previous studies have suggested that communism is positively correlated with interdependence. For example, social history research in Russia has suggested that citizens had high collectivist expression prior to the end of the Soviet Union when Russia was a communist country (Skrebyte et al., 2016). Another earlier study indicated that higher levels of interdependent self-construal are strongly correlated with ToM (Ray et al., 2010). In summary, ReHo in the MFG is negatively correlated with a belief in communism, indicating a higher level of interdependent self-construal. Furthermore, a belief in communism and ToM share the common component of interdependent self-construal.

In the rsFC analysis, a negative correlation between the BCS score and the strength of the functional connectivity between the left thalamus and the bilateral precuneus was identified. Previous studies in social neuroscience have also identified that precuneus is associated with ToM abilities. An fMRI study conducted by Gweon et al. (2012), which involved testing participants on a series of ToM tasks, found that precuneus was activated during the execution of specific tasks related to thinking about other thoughts of people; however, the connectivity between the thalamus and precuneus is less well-understood. Zhou et al. (2013) have suggested that an abnormal increase in the connectivity between the thalamus and specific brain areas, such as the precuneus, is an indication of cognition impairment. In that study, patients with Alzheimer's disease were compared to subjects with normal cognition, and a significant increase of functional connectivity was found between the bilateral thalamus and precuneus in the group with Alzheimer's disease. They concluded that this connectivity was likely a compensatory mechanism resulting from cognition impairment. Conversely, they suggested that decreased connectivity between the thalamus and bilateral precuneus could indicate that both are functioning well individually. Previous studies have suggested that both the thalamus and precuneus are key areas related to ToM (e.g., Rilling et al., 2004). Furthermore, the results of the mediation analysis in study 2 are consistent with such a conclusion. The connectivity between the thalamus and precuneus could partially negatively mediate the relationship between ReHo in the left thalamus and the BCS scores. Specifically, the lower the functional connectivity between the thalamus and precuneus, the stronger the relationship between the thalamus and a belief in communism.

To conclude, this study examined whether individual differences in the belief of participants in communism were associated with ToM abilities. At the behavioral level, we found a belief in communism to be significantly correlated with ToM abilities. At the neural level, a belief in communism was linked to core regions in the ToM network, such as the thalamus and the MFG. Taken together, these results enhance our understanding of the psychological basis of a belief in communism from a multi-disciplinary perspective.

## Limitations

This study has some limitations. First, only the relationship between a belief in communism and ToM was investigated. The relationship between ToM and other types of beliefs should also be explored. Second, the scale used to assess a belief in communism is not well-established; therefore, further studies should consider formally revising the scale. Third, this study is fundamentally correlational; therefore, a causal study of the relationship between a belief in communism and ToM should be conducted in the future to confirm these findings.

## Data Availability Statement

The questionnaires and dataset for study 1 can be found in the OSF link: https://osf.io/9azuk/?view_only=97dcc6b5c0ae47babb930b75952d5ad1. The raw data for study 2 will be made available by the authors, without undue reservation.

## Ethics Statement

The studies involving human participants were reviewed and approved by Human Research Ethics Committee for Non-Clinical Faculties, School of Psychology, South China Normal University. The patients/participants provided their written informed consent to participate in this study.

## Author Contributions

JC designed the research study. OC collected and analyzed the data and wrote the manuscript. FG proofread the manuscript and provided guidance on the data analysis section. YS, YD, and HY helped to collect the data and organized the raw data. All authors contributed to the article and approved the submitted version.

## Conflict of Interest

The authors declare that the research was conducted in the absence of any commercial or financial relationships that could be construed as a potential conflict of interest.

## Publisher's Note

All claims expressed in this article are solely those of the authors and do not necessarily represent those of their affiliated organizations, or those of the publisher, the editors and the reviewers. Any product that may be evaluated in this article, or claim that may be made by its manufacturer, is not guaranteed or endorsed by the publisher.

## References

[B1] Aketa H. (1999). Structure and measurement of empathy: Japanese version of Davis's Interpersonal Reactivity Index (IRI-J). Psychol. Rep. Sophia Univ. 2, 19–31.

[B2] Arous A. Mrizak J. Trabelsi R. Aissa A. Ammar H. B. El Hechmi Z. (2016). How do social cognition dimensions relate to DSM-5 dimensions of psychosis?. Eur. Psychiatry 33, S285–S286. 10.1016/j.eurpsy.2016.01.607

[B3] Astington J. W. Jenkins J. M. (1995). Theory of mind development and social understanding. Cogn. Emot. 9, 151–165. 10.1080/0269993950840900627780091

[B4] Biswal B. Zerrin Yetkin F. Haughton V. M. Hyde J. S. (1995). Functional connectivity in the motor cortex of resting human brain using echo-planar MRI. Magn. Reson. Med. 34, 537–541. 10.1002/mrm.1910340409 8524021

[B5] Bukharin N. Preobrazhenskii E. A. (1921). ABC of Communism. Glasgow: Socialist Labour Press.

[B6] Chen X. Lu B. Yan C. G. (2018). Reproducibility of r-fmri metrics on the impact of different strategies for multiple comparison correction and sample sizes. Hum. Brain Mapp. 39, 300–318. 10.1002/hbm.23843 29024299 PMC6866539

[B7] Decety J. Lamm C. (2007). The role of the right temporoparietal junction in social interaction: how low-level computational processes contribute to meta-cognition. Neuroscientist 13, 580–593. 10.1177/1073858407304654 17911216

[B8] Elliott R. Völlm B. Drury A. McKie S. Richardson P. William Deakin J. F. (2006). Co-operation with another player in a financially rewarded guessing game activates regions implicated in theory of mind. Soc. Neurosci. 1, 385–395. 10.1080/17470910601041358 18633801

[B9] Friston K. J. Williams S. Howard R. Frackowiak R. S. Turner R. (1996). Movement-related effects in fMRI time-series. Magn. Reson. Med. 35, 346–355. 10.1002/mrm.1910350312 8699946

[B10] Gavreliuc A. Ciobotă C. I. (2013). Culture and self-construal: implications for the social cognitions of young cohorts in Romania. Proc. Soc. Behav. Sci. 78, 270–274. 10.1016/j.sbspro.2013.04.293

[B11] Gweon H. Dodell-Feder D. Bedny M. Saxe R. (2012). Theory of mind performance in children correlates with functional specialization of a brain region for thinking about thoughts. Child Dev. 83, 1853–1868. 10.1111/j.1467-8624.2012.01829.x 22849953

[B12] Hayes A. F. (2013). Introduction to Mediation, Moderation, and Conditional Process Analysis: A Regression-Based Approach. New York, NY: Guilford Press.

[B13] Hughes C. Leekam S. (2004). What are the links between theory of mind and social relations? Review, reflections and new directions for studies of typical and atypical development. Soc. Dev. 13, 590–619. 10.1111/j.1467-9507.2004.00285.x

[B14] Kana R. K. Maximo J. O. Williams D. L. Keller T. A. Schipul S. E. Cherkassky V. L.. (2015). Aberrant functioning of the theory-of-mind network in children and adolescents with autism. Mol. Autism 6:59. 10.1186/s13229-015-0052-x 26512314 PMC4624365

[B15] Kurian G. T. Boryczka J. M. (2010). Encyclopedia of Political Science. Washington, DC: CQ Press. 10.4135/9781608712434

[B16] Li L. M. W. Luo S. Ma J. Lin Y. Fan L. Zhong S.. (2018). Functional connectivity pattern underlies individual differences in independent self-construal. Soc. Cogn. Affect. Neurosci. 13, 269–280. 10.1093/scan/nsy008 29385622 PMC5836281

[B17] Lissek S. Peters S. Fuchs N. Witthaus H. Nicolas V. Tegenthoff M.. (2008). Cooperation and deception recruit different subsets of the theory-of-mind network [Journal Article; Research Support, Non-U.S. Gov't]. PLoS ONE 3:e2023. 10.1371/journal.pone.0002023 18431500 PMC2295259

[B18] Little R. J. Rubin D. B. (2002). Statistical Analysis With Missing Data, 2nd Edn. New Jersey: Wiley. 10.1002/9781119013563

[B19] Liu Y. Wu B. Wang X. Li W. Zhang T. Wu X.. (2017). Oxytocin effects on self-referential processing: behavioral and neuroimaging evidence. Soc. Cogn. Affect. Neurosci. 12, 1845–1858. 10.1093/scan/nsx116 29040763 PMC5716198

[B20] McFarland S. (1998). Communism as religion. Int. J. Psychol. Relig. 8, 33–48. 10.1207/s15327582ijpr0801_5

[B21] Paretskaya A. (2010). The Soviet communist party and the other spirit of capitalism. Sociol. Theory 28, 377–401. 10.1111/j.1467-9558.2010.01382.x

[B22] Premack D. Woodruff G. (1978). Does the chimpanzee have a theory of mind?. Behav. Brain Sci. 1, 515–526. 10.1017/S0140525X00076512

[B23] Proverbio A. M. Riva F. Paganelli L. Cappa S. F. Canessa N. Perani D.. (2011). Neural coding of cooperative vs. affective human interactions: 150 ms to code the action's purpose. PLoS ONE 6:e22026. 10.1371/journal.pone.0022026 21760948 PMC3131384

[B24] Ray R. D. Shelton A. L. Hollon N. G. Matsumoto D. Frankel C. B. Gross J. J.. (2010). Interdependent self-construal and neural representations of self and mother. Soc. Cogn. Affect. Neurosci. 5, 318–32039. 10.1093/scan/nsp039 19822601 PMC2894675

[B25] Reniers R. L. Völlm B. A. Elliott R. Corcoran R. (2014). Empathy, ToM, and self–other differentiation: an fMRI study of internal states. Soc. Neurosci. 9, 50–62. 10.1080/17470919.2013.861360 24294841

[B26] Reniers R. L. E. P. Corcoran R. Drake R. Shryane N. M. Völlm B. A. (2011). The qcae: a questionnaire of cognitive and affective empathy. J. Pers. Assess. 93, 84–95. 10.1080/00223891.2010.528484 21184334

[B27] Rilling J. K. Sanfey A. G. Aronson J. A. Nystrom L. E. Cohen J. D. (2004). The neural correlates of theory of mind within interpersonal interactions. Neuroimage 22, 1694–1703. 10.1016/j.neuroimage.2004.04.015 15275925

[B28] Sally D. Hill E. (2006). The development of interpersonal strategy: autism, theory-of-mind, cooperation and fairness. J. Econom. Psychol. 27, 73–97.

[B29] Schilbach L. Eickhoff S. B. Rotarska-Jagiela A. Fink G. R. Vogeley K. (2008). Minds at rest? Social cognition as the default mode of cognizing and its putative relationship to the “default system” of the brain. Conscious. Cogn. 17, 457–467. 10.1016/j.concog.2008.03.013 18434197

[B30] Shamay-Tsoory S. G. Tibi-Elhanany Y. Aharon-Peretz J. (2006). The ventromedial prefrontal cortex is involved in understanding affective but not cognitive Theory of Mind stories. Soc. Neurosci. 1, 149–166. 10.1080/17470910600985589 18633784

[B31] Skrebyte A. Garnett P. Kendal J. R. (2016). Temporal relationships between individualism–collectivism and the economy in Soviet Russia: a word frequency analysis using the Google Ngram corpus. J. Cross Cult. Psychol. 47, 1217–1235. 10.1177/0022022116659540

[B32] Špiláková B. Shaw D. J. Czekóová K. Brázdil M. (2019). Dissecting social interaction: dual-fMRI reveals patterns of interpersonal brain-behavior relationships that dissociate among dimensions of social exchange. Soc. Cogn. Affect. Neurosci. 14, 225–235. 10.1093/scan/nsz004 30649548 PMC6374606

[B33] Wang X. (2002). The post-communist personality: the spectre of China's capitalist market reforms. China J. 47, 1–17. 10.2307/3182071

[B34] Wang X. S. Su Y. J. (2019). Revision of QCAE empathy scale for Chinese adolescents. Psychology 7, 536–547. 10.16842/j.cnki.issn2095-5588.2019.09.003

[B35] Worsley K. J. Macdonald D. Cao J. Shafie K. Evans A. C. (1996). Statistical analysis of cortical surfaces. Neuroimage 3:S108. 10.1016/S1053-8119(96)80110-8

[B36] Xiang Y. Kong F. Wen X. Wu Q. Mo L. (2016). Neural correlates of envy: regional homogeneity of resting-state brain activity predicts dispositional envy. Neuroimage 142, 225–230. 10.1016/j.neuroimage.2016.08.003 27498369

[B37] Yan C. Zang Y. (2010). DPARSF: a MATLAB toolbox for “pipeline” data analysis of resting-state fMRI. Front. Syst. Neurosci. 4:13. 10.3389/fnsys.2010.00013 20577591 PMC2889691

[B38] Yan C. G. Wang X. D. Zuo X. N. Zang Y. F. (2016). DPABI: data processing and analysis for (resting-state) brain imaging. Neuroinformatics 14, 339–351. 10.1007/s12021-016-9299-4 27075850

[B39] Zalta E. N. Nodelman U. Allen C. (2008). The Stanford Encyclopedia of Philosophy. Palo Alto, CA: Stanford University.

[B40] Zang Y. Jiang T. Lu Y. He Y. Tian L. (2004). Regional homogeneity approach to fMRI data analysis. Neuroimage 22, 394–400. 10.1016/j.neuroimage.2003.12.030 15110032

[B41] Zhang F. Dong Y. Wang K. (2010). Reliability and validity of the Chinese version of the Interpersonal Reactivity Index-C. Chin. J. Clin. Psychol. 18, 155–157. Available online at: http://clinicalpsychojournal.yywkt.cn/Magazine/Show.aspx?ID=139326

[B42] Zhou B. Liu Y. Zhang Z. An N. Yao H. Wang P.. (2013). Impaired functional connectivity of the thalamus in Alzheimer's disease and mild cognitive impairment: a resting-state fMRI study. Curr. Alzheimer Res. 10, 754–766. 10.2174/15672050113109990146 23905993

